# Solvate-free bis­(triphenylphosphine)iminium chloride

**DOI:** 10.1107/S1600536810046325

**Published:** 2010-11-17

**Authors:** Carsten Knapp, Rabiya Uzun

**Affiliations:** a Institut für Anorganische und Analytische Chemie, Albert-Ludwigs-Universität Freiburg, Albertstrasse 21, 79104 Freiburg i. Br., Germany

## Abstract

The title compound, C_36_H_30_NP_2_^+^·Cl^−^, crystallized in the solvate-free form from a CH_3_CN/OEt_2_ solution. The chloride anion and the N atom of the [(Ph_3_P)_2_N]^+^ cation are located on a twofold axis, yielding overall symmetry 2 for the cation. The central P—N—P angle [133.0 (3)°] is at the low end of the range of observed P—N—P angles.

## Related literature

Several bis­(triphenyl­phosphine)iminium chloride structures containing solvate mol­ecules have been determined. For [(Ph_3_P)_2_N]Cl·B(OH)_3_, see: Andrews *et al.* (1983 ▶); for [(Ph_3_P)_2_N]Cl·CH_3_C_6_H_5_, see: Weller *et al.* (1993 ▶); for [(Ph_3_P)_2_N]Cl·CH_2_Cl_2_, see: Carroll *et al.* (1996 ▶); for [(Ph_3_P)_2_N]Cl·CH_2_Cl_2_·H_2_O, see: de Arellano (1997 ▶). Other bis­(triphenyl­phosphine)iminium halide structures have been determined: for [(Ph_3_P)_2_N]Br·CH_3_CN, see: Knapp & Uzun (2010 ▶); for [(Ph_3_P)_2_N]I, see: Beckett *et al.* (2010 ▶). For a discussion of the [(Ph_3_P)_2_N]^+^ cation, see: Lewis & Dance (2000 ▶). For a description of the Cambridge Structural Database, see: Allen (2002 ▶). For the synthesis, see: Ruff & Schlientz (1974 ▶).
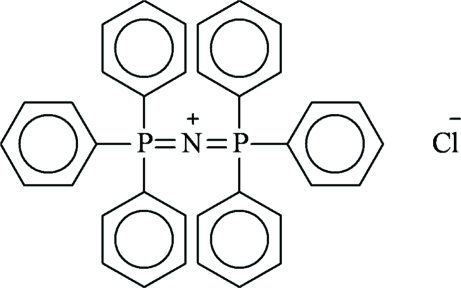


## Experimental

### 

#### Crystal data

C_36_H_30_NP_2_^+^·Cl^−^
*M* *_r_* = 574.00
Monoclinic, 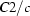

*a* = 15.094 (3) Å
*b* = 10.499 (2) Å
*c* = 18.615 (4) Å
β = 99.06 (3)°
*V* = 2913.0 (10) Å^3^
*Z* = 4
Mo *K*α radiation
μ = 0.27 mm^−1^
*T* = 123 K
0.30 × 0.23 × 0.23 mm

#### Data collection

Rigaku R-AXIS Spider diffractometer
Absorption correction: multi-scan (*ABSCOR*; Higashi, 2001 ▶) *T*_min_ = 0.924, *T*_max_ = 0.941
7362 measured reflections
2551 independent reflections
2296 reflections with *I* > 2σ(*I*)
*R*_int_ = 0.043

#### Refinement

*R*[*F* ^2^ > 2σ(*F* ^2^)] = 0.049
*wR*(*F* ^2^) = 0.135
*S* = 1.24
2551 reflections
183 parameters
H-atom parameters constrained
Δρ_max_ = 0.41 e Å^−3^
Δρ_min_ = −0.42 e Å^−3^

### 

Data collection: *CrystalClear* (Rigaku, 2007 ▶); cell refinement: *CrystalClear*; data reduction: *CrystalClear*; program(s) used to solve structure: *SHELXS97* (Sheldrick, 2008 ▶); program(s) used to refine structure: *SHELXL97* (Sheldrick, 2008 ▶); molecular graphics: *DIAMOND* (Brandenburg & Putz, 2010 ▶); software used to prepare material for publication: *SHELXL97*.

## Supplementary Material

Crystal structure: contains datablocks I, global. DOI: 10.1107/S1600536810046325/fi2099sup1.cif

Structure factors: contains datablocks I. DOI: 10.1107/S1600536810046325/fi2099Isup2.hkl

Additional supplementary materials:  crystallographic information; 3D view; checkCIF report

## Figures and Tables

**Table:** 

P1—N1	1.5984 (18)
P1—C7	1.795 (3)
P1—C1	1.802 (3)
P1—C13	1.811 (3)

**Table:** 

P1—N1—P1^i^	133.0 (3)

Symmetry code: (i) 
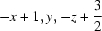
.

**Fig. 1.**
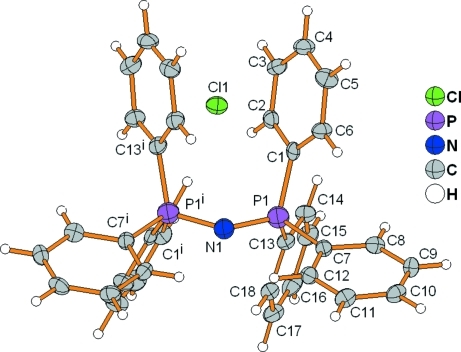
View of the ionic unit of [(Ph3P)2N]Cl. Displacement ellipsoids are shown at the 50% probability level and hydrogen atoms are drawn with arbitrary radii. Symmetry code: (i) 1-x, y, 1.5-z.

**Fig. 2.**
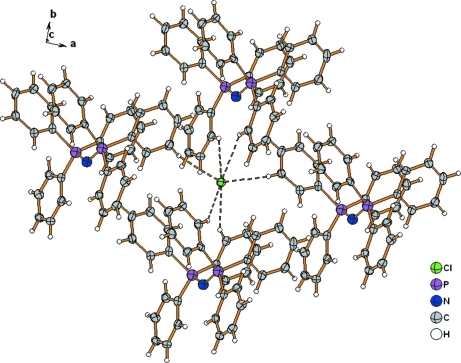
View of the surrounding of the chloride anion.

## supplementary crystallographic information

### Comment

The title compound [(Ph_3_P)_2_N]Cl ([PNP]Cl) is a very important starting material and numerous crystal structures containing the [(Ph_3_P)_2_N]^+^ cation are known. The Cambridge Structural Database (Allen, 2002) currently contains more than 1200 structures containing the [(Ph_3_P)_2_N]^+^ cation. Usually this cation is partnered by a bulky cation, while crystal structures containing small anions and especially halides are rare. Very recently, the crystal structures of solvate-free [(Ph_3_P)_2_N]I (Beckett *et al.*, 2010) and [(Ph_3_P)_2_N]Br^.^CH_3_CN (Knapp *et al.*, 2010) were published.

Several crystal structures of [(Ph_3_P)_2_N]Cl containing solvate molecules have been determined, *e.g.* [(Ph_3_P)_2_N]Cl^.^B(OH)_3_ (Andrews *et al.* (1983)), [(Ph_3_P)_2_N]Cl^.^CH_3_C_6_H_5_, (Weller *et al.* (1993)), [(Ph_3_P)_2_N]Cl^.^CH_2_Cl_2_ (Carroll *et al.* (1996)), [(Ph_3_P)_2_N]Cl^.^CH_2_Cl_2_^.^H_2_O (de Arellano (1997)). Surprisingly, the crystal structure of the parent compound [(Ph_3_P)_2_N]Cl was still unknown.

[(Ph_3_P)_2_N]Cl has been synthesized according to a published method (Ruff *et al.*, 1974) and solvate-free single crystals suitable for X-ray diffraction were obtained by layering a CH_3_CN solution with diethyl ether. The chlorine anion and the [(Ph_3_P)_2_N]^+^ cation are located on a 2 axis, yielding overall symmetry 2 of the cation. The central P—N—P angle [133.1 (3)°] is on the low end of the range of observed P—N—P angles. The P-N (1.597 (2) Å) and P-C distances (179.3 (4)–180.8 (4) Å) are in the expected range.

### Experimental

[(Ph_3_P)_2_N]Cl has been synthesized according to a published method (Ruff *et al.*, 1974). Single crystals suitable for X-ray diffraction were obtained by layering a CH_3_CN solution with diethyl ether.

### Refinement

The hydrogen atoms were positioned geometrically and refined using a riding model. The same *U_iso_* value was used for all H atoms, which refined to 0.031 (3) Å^2^.

### Crystal data

**Table:**

C_36_H_30_NP_2_^+^·Cl^−^	*F*(000) = 1200
*M**_r_* = 574.00	*D*_x_ = 1.309 Mg m^−^^3^
Monoclinic, *C*2/*c*	Mo *K*α radiation, λ = 0.71073 Å
Hall symbol: -C 2yc	Cell parameters from 1435 reflections
*a* = 15.094 (3) Å	θ = 2.2–27.5°
*b* = 10.499 (2) Å	µ = 0.27 mm^−^^1^
*c* = 18.615 (4) Å	*T* = 123 K
β = 99.06 (3)°	Block, colourless
*V* = 2913.0 (10) Å^3^	0.30 × 0.23 × 0.23 mm
*Z* = 4	

### Data collection

**Table:**

Rigaku R-AXIS Spider diffractometer	2551 independent reflections
Radiation source: sealed tube	2296 reflections with *I* > 2σ(*I*)
graphite	*R*_int_ = 0.043
Detector resolution: 10.0000 pixels mm^-1^	θ_max_ = 25.0°, θ_min_ = 2.2°
ω scans and/or φ scans	*h* = −17→17
Absorption correction: multi-scan (*ABSCOR*; Higashi, 2001)	*k* = −12→11
*T*_min_ = 0.924, *T*_max_ = 0.941	*l* = −20→22
7362 measured reflections	

### Refinement

**Table:**

Refinement on *F*^2^	Primary atom site location: structure-invariant direct methods
Least-squares matrix: full	Secondary atom site location: difference Fourier map
*R*[*F*^2^ > 2σ(*F*^2^)] = 0.049	Hydrogen site location: inferred from neighbouring sites
*wR*(*F*^2^) = 0.135	H-atom parameters constrained
*S* = 1.24	*w* = 1/[σ^2^(*F*_o_^2^) + 10.5312*P*] where *P* = (*F*_o_^2^ + 2*F*_c_^2^)/3
2551 reflections	(Δ/σ)_max_ < 0.001
183 parameters	Δρ_max_ = 0.41 e Å^−^^3^
0 restraints	Δρ_min_ = −0.42 e Å^−^^3^

### Special details

**Table:**

Geometry. All e.s.d.'s (except the e.s.d. in the dihedral angle between two l.s. planes) are estimated using the full covariance matrix. The cell e.s.d.'s are taken into account individually in the estimation of e.s.d.'s in distances, angles and torsion angles; correlations between e.s.d.'s in cell parameters are only used when they are defined by crystal symmetry. An approximate (isotropic) treatment of cell e.s.d.'s is used for estimating e.s.d.'s involving l.s. planes.
Refinement. Refinement of *F*^2^ against ALL reflections. The weighted *R*-factor *wR* and goodness of fit *S* are based on *F*^2^, conventional *R*-factors *R* are based on *F*, with *F* set to zero for negative *F*^2^. The threshold expression of *F*^2^ > 2σ(*F*^2^) is used only for calculating *R*-factors(gt) *etc*. and is not relevant to the choice of reflections for refinement. *R*-factors based on *F*^2^ are statistically about twice as large as those based on *F*, and *R*- factors based on ALL data will be even larger.

### Fractional atomic coordinates and isotropic or equivalent isotropic displacement parameters (Å^2^)

**Table:**

	*x*	*y*	*z*	*U*_iso_*/*U*_eq_	
Cl1	0.5000	0.80128 (11)	0.7500	0.0264 (3)	
P1	0.53916 (5)	0.32003 (8)	0.82724 (4)	0.0175 (2)	
N1	0.5000	0.2594 (4)	0.7500	0.0200 (8)	
C1	0.45695 (19)	0.4133 (3)	0.86442 (16)	0.0177 (6)	
C2	0.4358 (2)	0.5349 (3)	0.83690 (17)	0.0206 (7)	
H2	0.4700	0.5724	0.8038	0.030 (3)*	
C3	0.3645 (2)	0.6011 (3)	0.85815 (19)	0.0262 (8)	
H3	0.3505	0.6844	0.8399	0.030 (3)*	
C4	0.3135 (2)	0.5461 (4)	0.90596 (19)	0.0293 (8)	
H4	0.2639	0.5908	0.9193	0.030 (3)*	
C5	0.3353 (2)	0.4258 (3)	0.9342 (2)	0.0288 (8)	
H5	0.3009	0.3891	0.9675	0.030 (3)*	
C6	0.4070 (2)	0.3582 (3)	0.91427 (18)	0.0258 (7)	
H6	0.4220	0.2761	0.9340	0.030 (3)*	
C7	0.57029 (19)	0.1897 (3)	0.88839 (17)	0.0182 (7)	
C8	0.6079 (2)	0.2160 (3)	0.96055 (18)	0.0247 (7)	
H8	0.6183	0.3018	0.9758	0.030 (3)*	
C9	0.6297 (2)	0.1184 (3)	1.00954 (18)	0.0260 (7)	
H9	0.6548	0.1365	1.0585	0.030 (3)*	
C10	0.6148 (2)	−0.0064 (3)	0.98669 (19)	0.0261 (8)	
H10	0.6298	−0.0738	1.0204	0.030 (3)*	
C11	0.5784 (2)	−0.0344 (3)	0.91551 (19)	0.0242 (7)	
H11	0.5685	−0.1204	0.9005	0.030 (3)*	
C12	0.5563 (2)	0.0643 (3)	0.86618 (18)	0.0224 (7)	
H12	0.5316	0.0457	0.8171	0.030 (3)*	
C13	0.6391 (2)	0.4148 (3)	0.82560 (17)	0.0216 (7)	
C14	0.6520 (2)	0.5357 (3)	0.85535 (18)	0.0232 (7)	
H14	0.6081	0.5726	0.8804	0.030 (3)*	
C15	0.7303 (2)	0.6030 (3)	0.8482 (2)	0.0299 (8)	
H15	0.7385	0.6868	0.8674	0.030 (3)*	
C16	0.7957 (2)	0.5487 (4)	0.8137 (2)	0.0314 (9)	
H16	0.8488	0.5950	0.8098	0.030 (3)*	
C17	0.7839 (2)	0.4266 (3)	0.78468 (19)	0.0275 (8)	
H17	0.8292	0.3888	0.7616	0.030 (3)*	
C18	0.7054 (2)	0.3602 (3)	0.78966 (18)	0.0249 (7)	
H18	0.6964	0.2777	0.7688	0.030 (3)*	

### Atomic displacement parameters (Å^2^)

**Table:**

	*U*^11^	*U*^22^	*U*^33^	*U*^12^	*U*^13^	*U*^23^
Cl1	0.0250 (6)	0.0205 (6)	0.0352 (7)	0.000	0.0095 (5)	0.000
P1	0.0157 (4)	0.0178 (4)	0.0194 (4)	0.0007 (3)	0.0037 (3)	0.0004 (3)
N1	0.0140 (17)	0.026 (2)	0.0199 (19)	0.000	0.0030 (14)	0.000
C1	0.0184 (15)	0.0189 (16)	0.0147 (14)	0.0012 (12)	−0.0006 (12)	−0.0041 (13)
C2	0.0217 (16)	0.0205 (17)	0.0194 (16)	−0.0022 (13)	0.0028 (13)	−0.0014 (14)
C3	0.0228 (16)	0.0240 (18)	0.0309 (18)	0.0045 (14)	0.0014 (14)	−0.0018 (15)
C4	0.0186 (16)	0.036 (2)	0.033 (2)	0.0056 (14)	0.0048 (14)	−0.0104 (17)
C5	0.0274 (18)	0.0265 (18)	0.036 (2)	−0.0023 (15)	0.0161 (15)	−0.0018 (17)
C6	0.0232 (17)	0.0279 (18)	0.0270 (18)	0.0008 (14)	0.0057 (14)	0.0038 (15)
C7	0.0161 (15)	0.0199 (16)	0.0192 (16)	0.0005 (12)	0.0044 (12)	0.0004 (13)
C8	0.0263 (17)	0.0210 (17)	0.0272 (18)	−0.0010 (14)	0.0055 (14)	0.0006 (15)
C9	0.0280 (18)	0.0304 (19)	0.0188 (16)	0.0008 (14)	0.0012 (13)	0.0025 (15)
C10	0.0230 (17)	0.0273 (18)	0.0287 (19)	0.0028 (14)	0.0062 (14)	0.0135 (15)
C11	0.0252 (17)	0.0151 (16)	0.0330 (19)	0.0000 (13)	0.0072 (14)	0.0033 (14)
C12	0.0177 (15)	0.0261 (18)	0.0236 (17)	−0.0016 (13)	0.0036 (13)	−0.0018 (15)
C13	0.0163 (15)	0.0246 (17)	0.0238 (17)	0.0001 (13)	0.0026 (12)	0.0033 (14)
C14	0.0235 (17)	0.0186 (16)	0.0276 (18)	−0.0014 (13)	0.0040 (13)	−0.0024 (14)
C15	0.0240 (17)	0.0280 (19)	0.036 (2)	−0.0052 (15)	−0.0003 (15)	−0.0018 (16)
C16	0.0163 (16)	0.043 (2)	0.033 (2)	−0.0046 (15)	−0.0021 (14)	0.0123 (17)
C17	0.0205 (16)	0.032 (2)	0.0305 (19)	0.0033 (14)	0.0050 (14)	0.0089 (16)
C18	0.0195 (16)	0.0311 (19)	0.0237 (17)	0.0026 (14)	0.0020 (13)	−0.0027 (15)

### Geometric parameters (Å, °)

**Table:**

P1—N1	1.5984 (18)	C8—H8	0.9500
P1—C7	1.795 (3)	C9—C10	1.385 (5)
P1—C1	1.802 (3)	C9—H9	0.9500
P1—C13	1.811 (3)	C10—C11	1.384 (5)
N1—P1^i^	1.5984 (18)	C10—H10	0.9500
C1—C2	1.394 (4)	C11—C12	1.390 (5)
C1—C6	1.409 (5)	C11—H11	0.9500
C2—C3	1.390 (5)	C12—H12	0.9500
C2—H2	0.9500	C13—C14	1.386 (5)
C3—C4	1.390 (5)	C13—C18	1.410 (4)
C3—H3	0.9500	C14—C15	1.401 (5)
C4—C5	1.387 (5)	C14—H14	0.9500
C4—H4	0.9500	C15—C16	1.383 (5)
C5—C6	1.392 (5)	C15—H15	0.9500
C5—H5	0.9500	C16—C17	1.392 (5)
C6—H6	0.9500	C16—H16	0.9500
C7—C12	1.386 (4)	C17—C18	1.391 (5)
C7—C8	1.401 (4)	C17—H17	0.9500
C8—C9	1.377 (5)	C18—H18	0.9500
			
N1—P1—C7	106.82 (17)	C8—C9—C10	119.3 (3)
N1—P1—C1	112.50 (12)	C8—C9—H9	120.3
C7—P1—C1	107.33 (15)	C10—C9—H9	120.3
N1—P1—C13	113.24 (13)	C11—C10—C9	121.1 (3)
C7—P1—C13	107.11 (14)	C11—C10—H10	119.5
C1—P1—C13	109.49 (15)	C9—C10—H10	119.5
P1—N1—P1^i^	133.0 (3)	C10—C11—C12	119.5 (3)
C2—C1—C6	120.2 (3)	C10—C11—H11	120.3
C2—C1—P1	119.2 (2)	C12—C11—H11	120.3
C6—C1—P1	120.2 (2)	C7—C12—C11	120.1 (3)
C3—C2—C1	119.7 (3)	C7—C12—H12	119.9
C3—C2—H2	120.1	C11—C12—H12	119.9
C1—C2—H2	120.1	C14—C13—C18	119.7 (3)
C4—C3—C2	120.4 (3)	C14—C13—P1	124.1 (2)
C4—C3—H3	119.8	C18—C13—P1	116.1 (3)
C2—C3—H3	119.8	C13—C14—C15	119.5 (3)
C5—C4—C3	119.9 (3)	C13—C14—H14	120.3
C5—C4—H4	120.0	C15—C14—H14	120.3
C3—C4—H4	120.0	C16—C15—C14	120.7 (3)
C4—C5—C6	120.7 (3)	C16—C15—H15	119.6
C4—C5—H5	119.6	C14—C15—H15	119.6
C6—C5—H5	119.6	C15—C16—C17	120.2 (3)
C5—C6—C1	119.0 (3)	C15—C16—H16	119.9
C5—C6—H6	120.5	C17—C16—H16	119.9
C1—C6—H6	120.5	C18—C17—C16	119.6 (3)
C12—C7—C8	119.5 (3)	C18—C17—H17	120.2
C12—C7—P1	121.5 (2)	C16—C17—H17	120.2
C8—C7—P1	118.9 (2)	C17—C18—C13	120.3 (3)
C9—C8—C7	120.5 (3)	C17—C18—H18	119.9
C9—C8—H8	119.8	C13—C18—H18	119.9
C7—C8—H8	119.8		
			
C7—P1—N1—P1^i^	−179.94 (11)	C12—C7—C8—C9	−0.9 (5)
C1—P1—N1—P1^i^	62.54 (12)	P1—C7—C8—C9	177.8 (2)
C13—P1—N1—P1^i^	−62.27 (13)	C7—C8—C9—C10	0.4 (5)
N1—P1—C1—C2	−76.9 (3)	C8—C9—C10—C11	0.1 (5)
C7—P1—C1—C2	165.9 (2)	C9—C10—C11—C12	−0.1 (5)
C13—P1—C1—C2	50.0 (3)	C8—C7—C12—C11	0.9 (5)
N1—P1—C1—C6	95.5 (3)	P1—C7—C12—C11	−177.8 (2)
C7—P1—C1—C6	−21.7 (3)	C10—C11—C12—C7	−0.4 (5)
C13—P1—C1—C6	−137.7 (3)	N1—P1—C13—C14	132.3 (3)
C6—C1—C2—C3	−0.8 (5)	C7—P1—C13—C14	−110.2 (3)
P1—C1—C2—C3	171.6 (2)	C1—P1—C13—C14	5.9 (3)
C1—C2—C3—C4	−0.8 (5)	N1—P1—C13—C18	−46.0 (3)
C2—C3—C4—C5	1.7 (5)	C7—P1—C13—C18	71.5 (3)
C3—C4—C5—C6	−1.0 (5)	C1—P1—C13—C18	−172.5 (2)
C4—C5—C6—C1	−0.5 (5)	C18—C13—C14—C15	1.1 (5)
C2—C1—C6—C5	1.4 (5)	P1—C13—C14—C15	−177.2 (3)
P1—C1—C6—C5	−170.9 (3)	C13—C14—C15—C16	−1.8 (5)
N1—P1—C7—C12	−2.0 (3)	C14—C15—C16—C17	0.8 (5)
C1—P1—C7—C12	118.9 (3)	C15—C16—C17—C18	1.0 (5)
C13—P1—C7—C12	−123.6 (3)	C16—C17—C18—C13	−1.7 (5)
N1—P1—C7—C8	179.3 (2)	C14—C13—C18—C17	0.7 (5)
C1—P1—C7—C8	−59.8 (3)	P1—C13—C18—C17	179.1 (3)
C13—P1—C7—C8	57.7 (3)		

Symmetry codes: (i) −*x*+1, *y*, −*z*+3/2.
